# Supplementary material for: pyProGA—A PyMOL plugin for protein residue network analysis
Source: PLoS One. 2021 Jul 30;16(7):e0255167. doi: 10.1371/journal.pone.0255167 (PMC8323899; doi:10.1371/journal.pone.0255167)
Supplement: S1 File — (PDF) [file pone.0255167.s001.pdf]

Supplementary information for:

pyProGA - A PyMOL plugin for protein residue network analysis

Vladimir Sladek[1,*], Yuta Yamamoto[2], Ryuhei Harada[3], Mitsuo Shoji[3], and Yasuteru Shigeta[3]

[1]Institute of Chemistry - Centre for Glycomics, Slovak Academy of Sciences, SK-845 38 Bratislava, Slovakia

[2]Department of Chemistry, Rikkyo University, Nishi-Ikebukuro, Toshima, Tokyo 171-8501, Japan

[3]Center for Computational Sciences, University of Tsukuba, Tsukuba, Ibaraki 305-8577, Japan

[*]correspondence to: sladek.vladimir@savba.sk

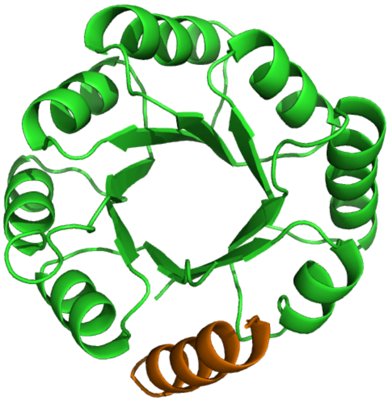

Figure S 1: The TIM barrel protein as used in our study. In the examples we construct a PIE-PRN model based on FMO calculations at DFTB level with the 3ob-3-1 parameter set as defined in the Gamess program package. One vertex/node n the PIE-PRN corresponds to one fragment in the FMO calculation. The FMO calculations yielded PIE energies between fragments used for edge creation. We apply the criterion $E_{\mathrm{tot}} \leq -1\,\mathrm{kcal\,mol^{-1}}$ to all types of bonds (incl. peptide) with the remaining settings kept at default. The sequence has the residue 17 missing, allowing us to formally treat the system as a dimer (complex) formed from the first barrel (orange colour) and the remainder of the sequence (green colour). This fact, as well as the fact that the string does have a beginning and end, does not allow us to call the structure symmetrical in a strict sense. However, for practical purpose we consider it to have a four-fold symmetry, as do the authors, who synthesized the molecule. The PyMOL object of the .pdb structure is called `structure` in pyProGA.

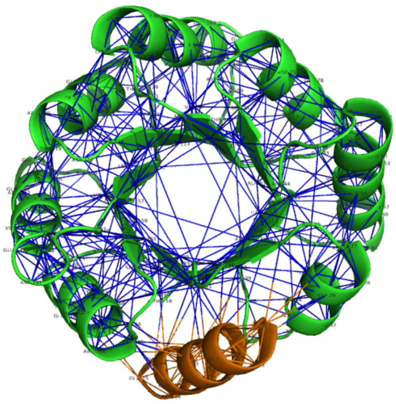

Figure S 2: A graphical representation of the PIE-PRN in PyMOL created with pyProGA. The lines are a separate object `3d_PEPRN`, here displayed together with the object `structure`. In this study we call the single helix (orange) monomer A and the rest (green) is monomer B. The graph of the PRN model of the dimer is labelled as G, and the monomer graphs as $G_{A(B)}$.

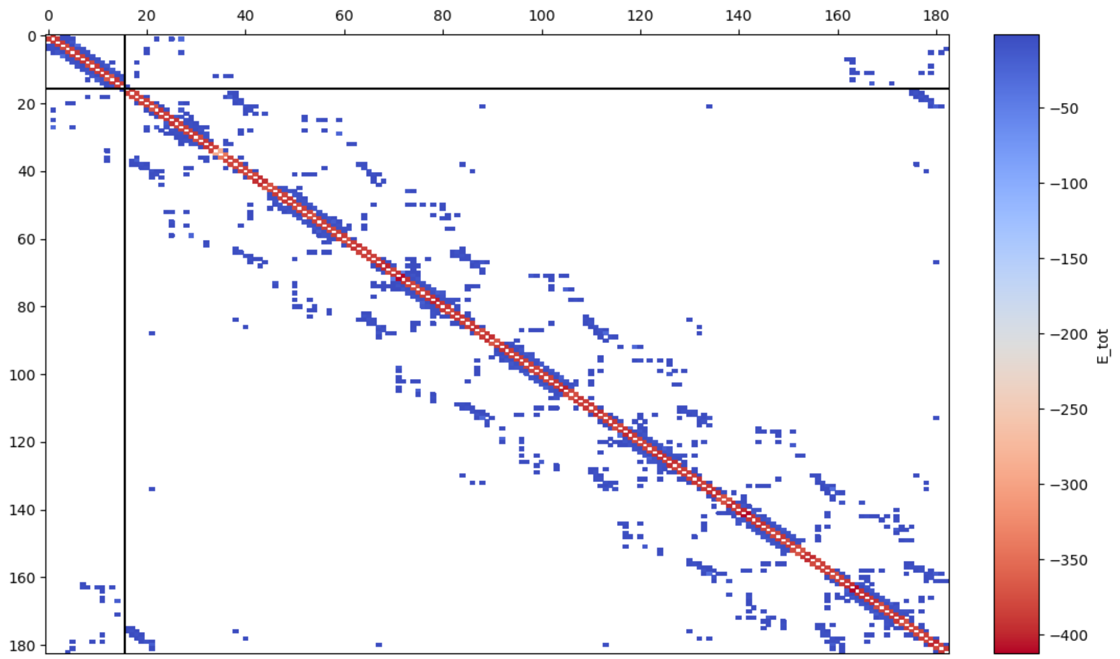

Figure S 3: A 2d map of the E_{tot} values for the fragment pairs that are connected by edges in G created in pyProGA. The map is clickable, enabling easy retrieval of node coordinates, name, and the value of the plotted quantity (here E_{tot}). The vertical and horizontal lines indicate monomer boundaries (their display is optional).
Customization and manipulation of the plot can be done in pyProGA *via* a standard Matplolib toolbar.

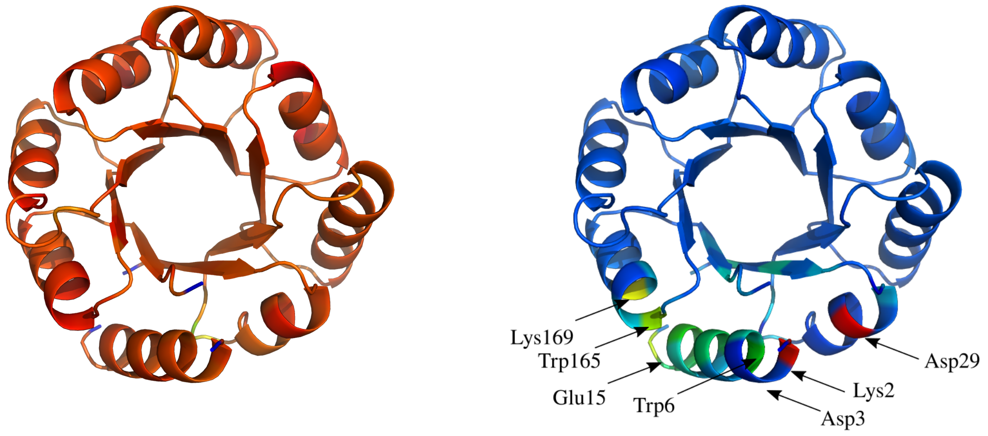

(a) Total energy centrality $C_k^{E_{\text{tot}}}(G)$ in PIE-PRN.

(b) Total energy centrality difference $\Delta C_k^{E_{\text{tot}}}$ in PIE-PRN.

Figure S 4: Total energy centrality $C_k^{E_{\text{tot}}}$ (a) in the dimer graph G does not show any significant variation of the magnitude. Only the terminal fragments have somewhat lover values. (b) the differential network analysis (NDA) of $C_k^{E_{\text{tot}}}$ shows that certain residues contribute more to the binding of the two monomers. Compare to the results in Fig. S 5b.

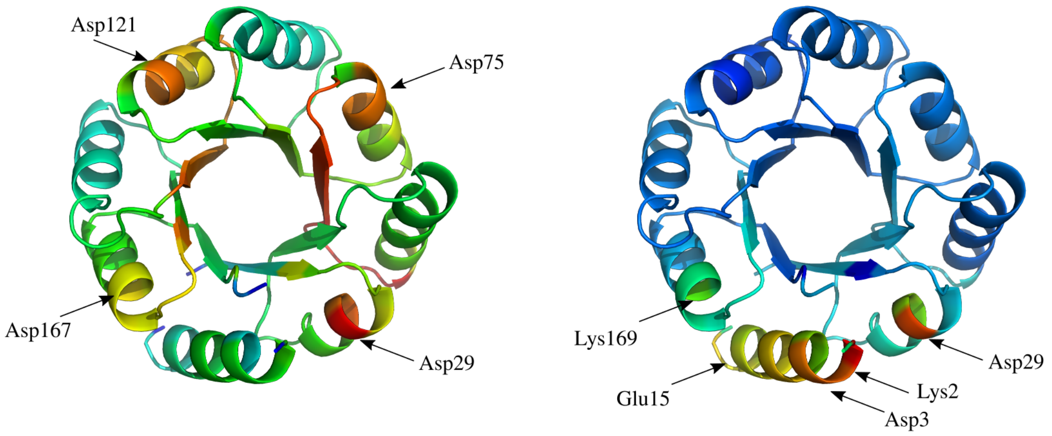

(a) Efficiency centrality $C_k^{\mathrm{eff}}(G)$ in PIE-PRN.

(b) Efficiency centrality difference $\Delta C_k^{\mathrm{eff}}$ in PIE-PRN. $\Delta C_k^{\mathrm{eff}} = C_k^{\mathrm{eff}}(G) - C_k^{\mathrm{eff}}(G_{A\cup B})$

Figure S 5: Efficiency centrality (a) in the dimer graph G does indicate that Asp29 is important for the small-world nature of this PRN model. We can see the four-fold symmetry of the TIM barrel protein rather clearly by the way how the ranking cyclically repeats it self in the sequence. Along Asp29, also Asp75, Asp121 and Asp167 score highly (amongst other residues). The score of Asp167 is somewhat lower than that of the others, owing to the fact that it is closer to the end of the sequence and to the (artificial) break of the sequence at residue 17. Contrary to that, Asp29 has a somewhat higher score, as it mediates also the contact of monomer A to monomer B *via* a strong interaction with Lys2. However, such centrality ranking does not reveal much about the interaction of monomer A and B (e.g. we do not see Lys2 score highly here). On the other hand, the efficiency centrality difference (b) paints a much clearer picture of which residues from the protein-protein interface are most contributing to the global efficiency of the dimer. Here we can clearly see that Lys2 ranks high.

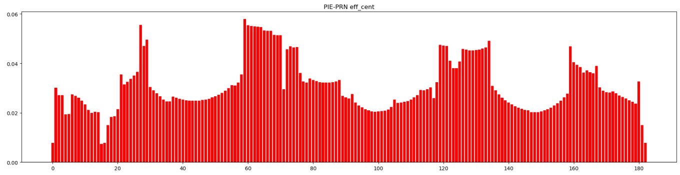

(a) Efficiency centrality C_k^{eff} in PIE-PRN.

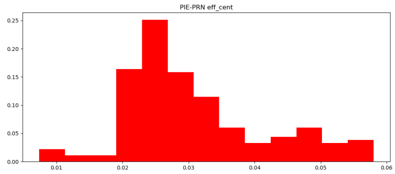

(b) Histogram of C_k^{eff} in PIE-PRN.

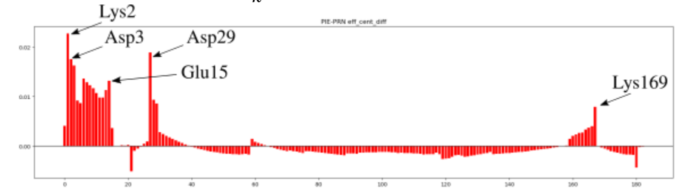

(c) Efficiency centrality difference ΔC_k^{eff} in PIE-PRN.

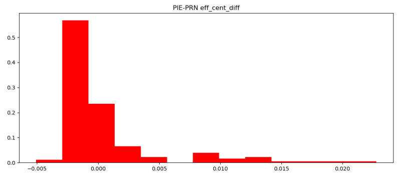

(d) Histogram of ΔC_k^{eff} in PIE-PRN.

Figure S 6: Bar plots and histograms made in pyProGA of C_k^{eff} and ΔC_k^{eff}. The bars in pyProGA are clickable to easily retrieve fragment/residue name and number and the height of the bar (plotted value of centrality). (a) in the dimer graph G we can, to some degree, see the four-fold symmetry of the TIM barrel protein by the way how the ranking cyclically repeats along the sequence. The fact, that the sequence has a break at position 17 and two open ends make it impossible to attain perfect symmetry. (b) The long tail on the right side in the C_k^{eff} histogram indicates that the network is of small-world character with few important vertices. (c) The bar plot of ΔC_k^{eff} allow deeper insight into which fragments gain higher C_k^{eff} ranking when the dimer forms. In other words, they are responsible for the stabilization of the complex. Some of them are specifically highlighted in Fig. S 5b as well. (d) Most fragments do not contribute to the stability of the dimer, hence their ΔC_k^{eff} score is (slightly) negative. The distribution has a long right-sided tail, indicating that few fragments contribute significantly to the dimer stabilization. Customization and manipulation of the plot can be done in pyProGA *via* a standard Matplolib toolbar.

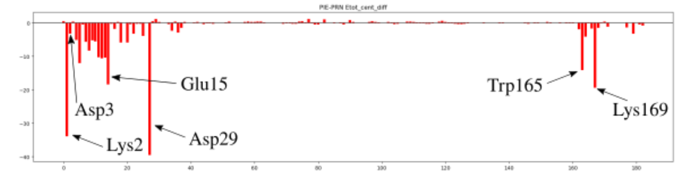

(a) Total energy centrality $C_k^{E_{\text{tot}}}(G)$ in PIE-PRN.

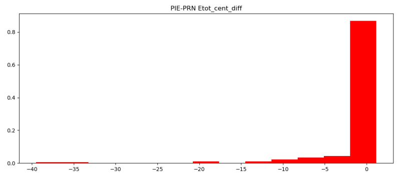

(b) Histogram of $C_k^{E_{\text{tot}}}(G)$ in PIE-PRN.

Figure S 7: Bar plot and histogram made in pyProGA of $C_k^{E_{\text{tot}}}(G)$. The correlation of $C_k^{E_{\text{tot}}}(G)$ and ΔC_k^{eff} in Fig. 6c is obvious for some fragments. A difference is seen for e.g. Asp3 that scores relatively highly in ΔC_k^{eff} but the actual change in E_{tot} is not as prominent. The reasons can be in principle twofold; first, Asp3 forms rather few interactions with fragments in monomer B. This explains the low $C_k^{E_{\text{tot}}}(G)$ score. The reason why Ala3 is picked up by ΔC_k^{eff} is a bit more convoluted. Asp3 is in monomer A, which is not connected to monomer B *via* covalent bonds. Monomer A has only 16 fragments/residues. Hence, the efficiency centrality of these fragments in the dimer graph G will not be very high, see Fig. 5a. However, Asp3 is adjacent to Lys2 *via* the strong peptide bond. The importance of Lys2 for binding between A and B is confirmed by its high $C_k^{E_{\text{tot}}}(G)$ (interaction with Asp29 is -28.5 kcal mol^{-1}) and ΔC_k^{eff} score. Asp3 is relatively central within monomer A, as it interacts strongly with Ala5 (-2.3 kcal mol^{-1}) and Trp6 (-9.1 kcal mol^{-1})†. By the combination of these facts (adjacency to Lys2 and centrality in monomer A), Asp3 becomes more central for the "small-worldedness" of the whole system. Hence is scores relatively highly in ΔC_k^{eff}.

† If we calculate C_k^{eff} in monomer A, we find that Asp3 is more central than Lys2.

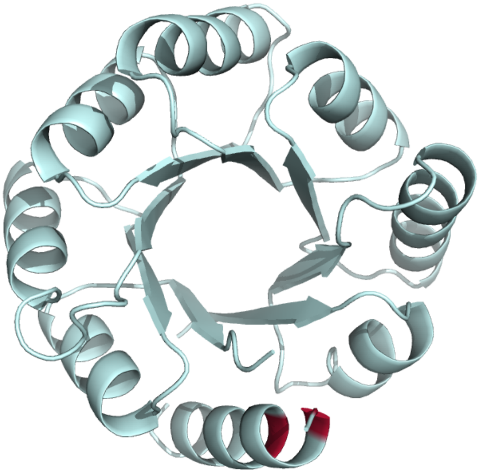

(a) PC 1: Lys2, Trp6 ($f^{(1)} = 0.21$).

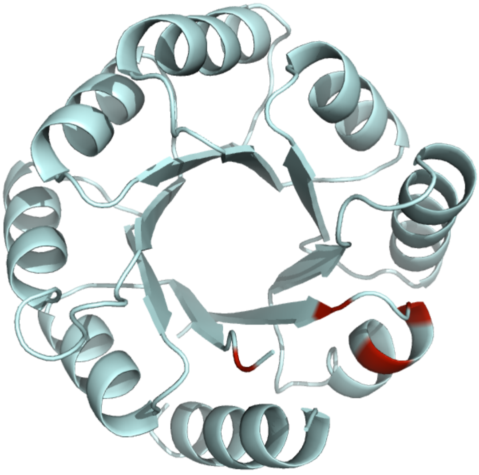

(b) PC 2: Ser24, Trp27, Asp29, Ala183 ($f^{(2)} = 0.41$).

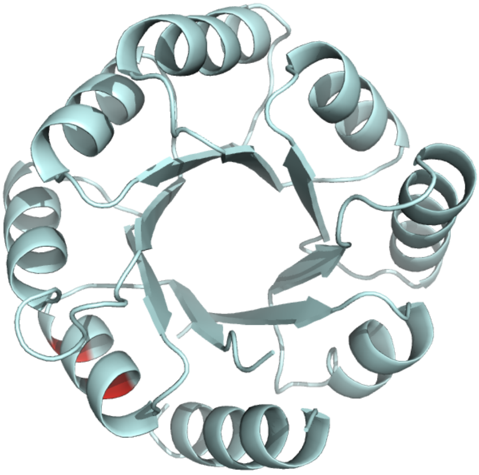

(c) PC 3: Lys169, Lys173 ($f^{(3)} = 0.53$).

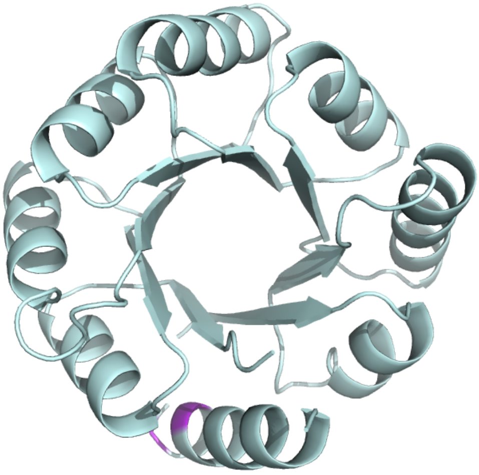

(d) PC 4: Leu12, Glu15 ($f^{(4)} = 0.64$).

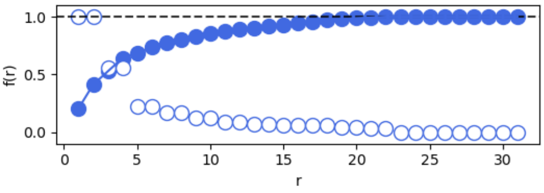

(e) Convergence of $f^{(r)}$ (full symbols) and normalised $\sigma_i/\max\{\sigma_i\}$ (empty symbols).

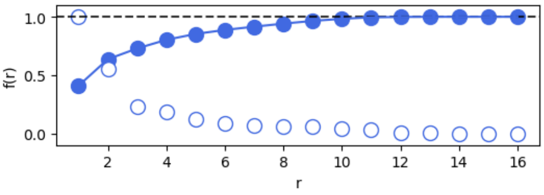

(f) Convergence of $f^{(r)}$ (full symbols) and normalised $\sigma_i/\max\{\sigma_i\}$ (empty symbols) in case if we analyse all interactions (attractive & repulsive) and do not use the adjacency matrix of G_{PPI}, rather the approach by Tanaka *et al.* **This is not used in the exemplar analysis in this work!**

Figure S 8: Principal components (motifs) from the SVD analysis of the PIE-PRN bipartite PPI. The vector component relevance was set to 0.01.

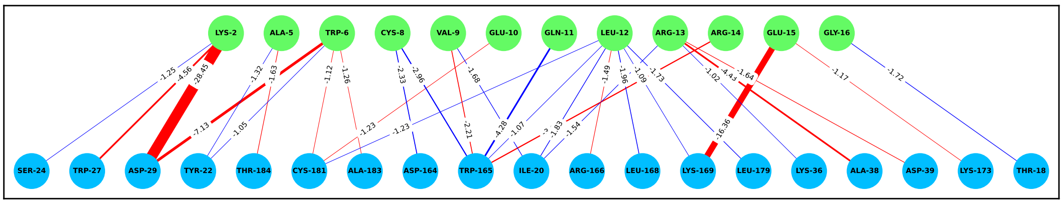

(a) Attractive interactions in the PPI.

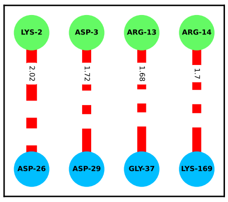

(b) Repulsive interactions in the PPI.

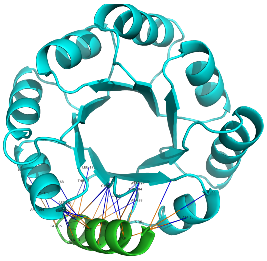

(c) 3d representation of attractive interactions in the PPI.

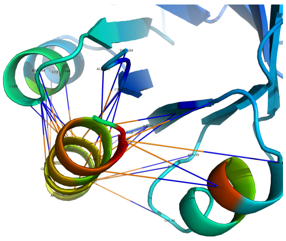

(d) Detail of the 3d representation of attractive interactions in the PPI. Residue colouring by $\Delta C_k^{\mathrm{eff}}$.

Figure S 9: The attractive and repulsive interactions constituting the bipartite PPI graph between monomer A (green) and B (blue). Thickness of edge in (a) and (b) corresponds to interaction strength, colour to character; red for prevailing electrostatic, blue for dispersion.

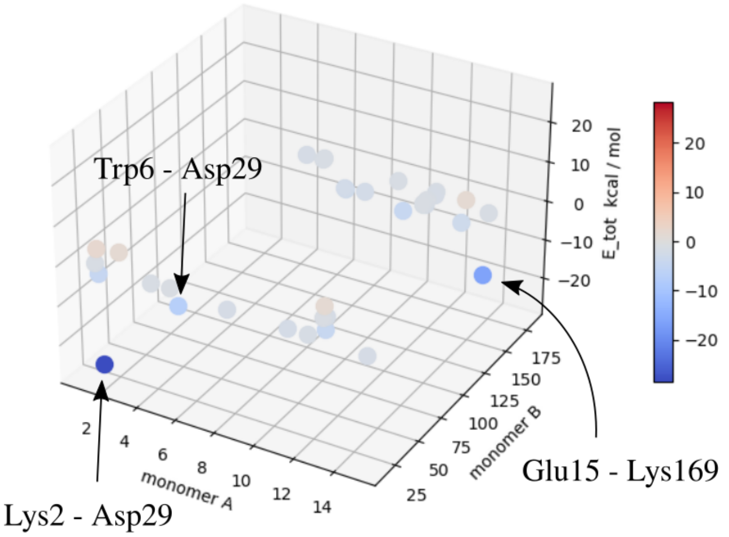

Figure S 10: The 3D-SPIE representation of the interactions in the PPI. The horizontal axes number fragments in the monomers A and B. The vertical axis (centred at zero) represents the energy component which was used for the creation of the graph. Colour gradient indicates magnitude of PIE.
The plot is clickable and can be manipulated (rotate, zoom, save, etc.) via a standard Matplotlib toolbar.

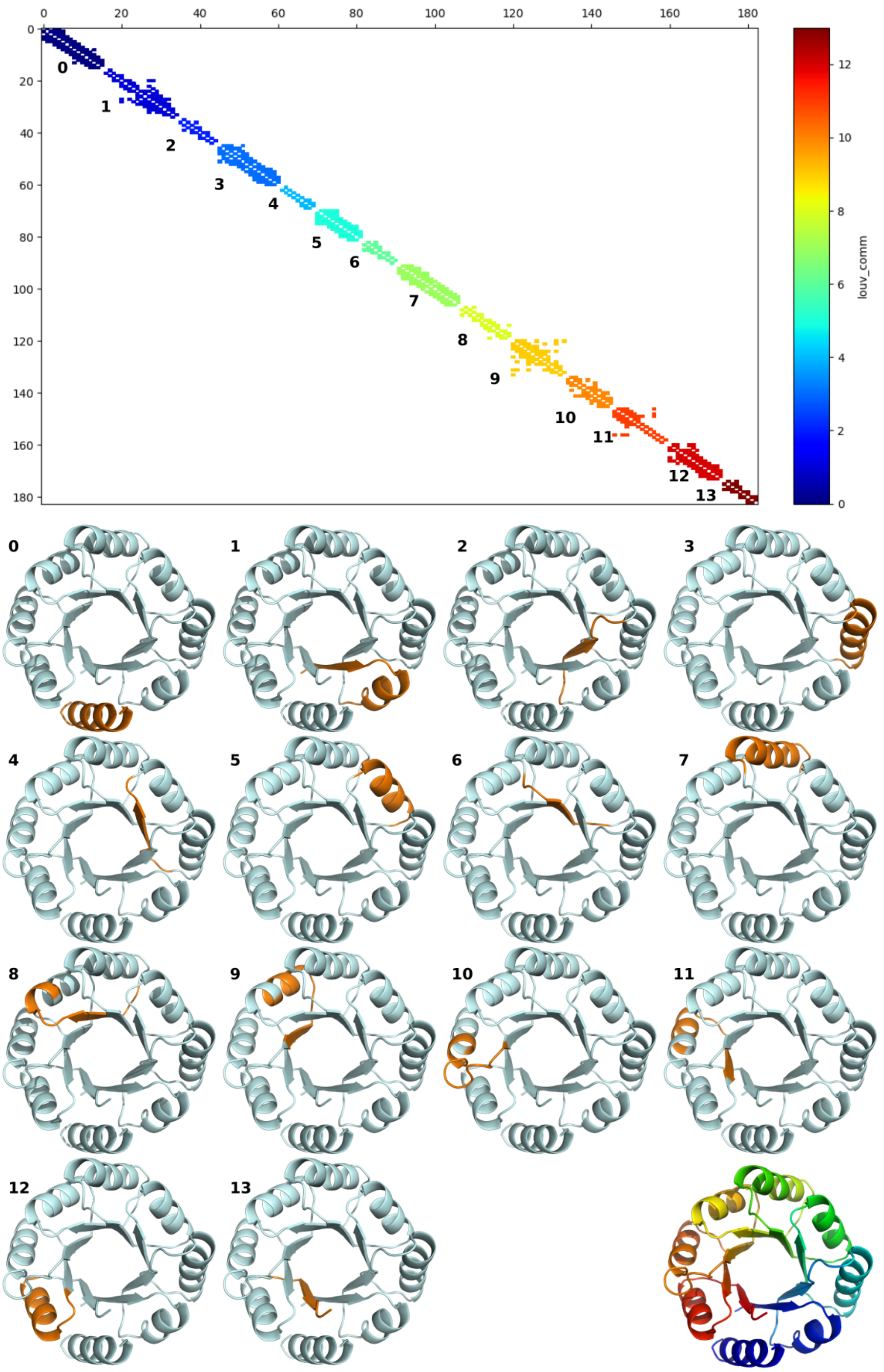

Figure S 11: Louvain communities in the PIE-PRN model. The top part depicts colour coded membership of nodes to communities using the '2D Edge Map' feature of pyProGA. The colour palette is the same as used in the right bottom structure. This is the representation where all communities are coloured at once, and is the default view in the PyMOL window after the calculation if finished. Since it may be difficult to see the partition borders clearly, pyProGA has a feature facilitating the highlight each partition (community, as seen here, or cluster). These are the fourteen structures with communities are highlighted in orange.

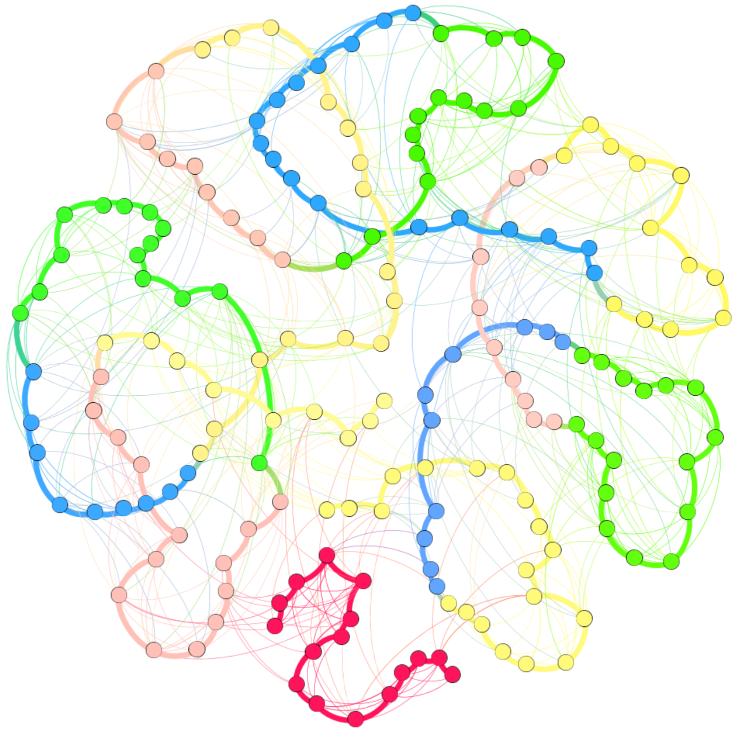

Figure S 12: Louvain communities in the PIE-PRN model. Graphic made in Gephi. The program features a variety of layout algorithms to position the nodes (here used is ForceAtlas2). Community colouring: red: 0; yellow: 1, 5, 9; blue: 2, 6, 10; green: 3, 7, 11; orange/pink: 4, 8, 12.
(The edge attribute `importance` must be copied to attribute `weight`, as Gephi interprets weight as the strength of an edge, rather than the cost (which is another standard interpretation we comply with in pyProGA) in order to achieve a similar layout. The settings can be checked in the .gephi file.).

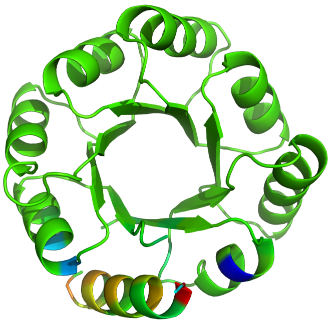

(**a**) Binding energies assigned to fragments in monomer A.

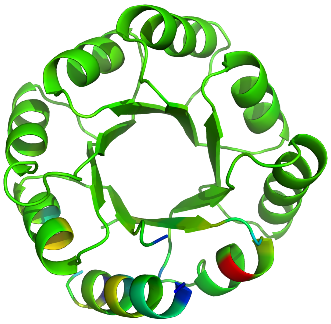

(**b**) Binding energies assigned to fragments in both monomers A and B.

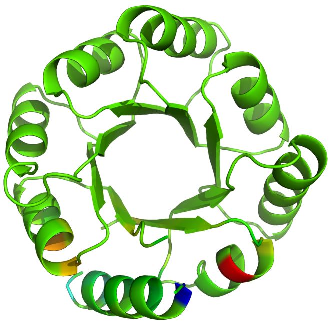

(**c**) Binding energies assigned to fragments in monomer B.

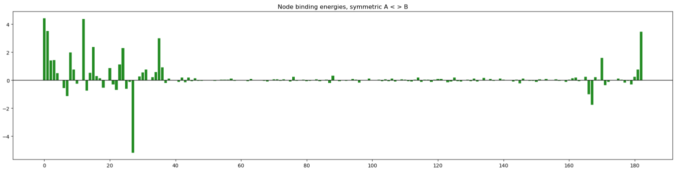

(**d**) Bar plot of binding energies assigned to fragments in both monomers A and B.

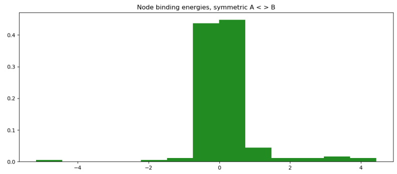

(**e**) Histogram of binding energies assigned to fragments in both monomers A and B.

Figure S 13: The binding energy between monomers A and B and different schemes of assignment to fragments. For the method and practical aspects of the technique see this D. Fedorov: manual for subsystem analysis.
