# Supplementary material for: pyProGA—A PyMOL plugin for protein residue network analysis
Source: PLoS One. 2021 Jul 30;16(7):e0255167. doi: 10.1371/journal.pone.0255167 (PMC8323899; doi:10.1371/journal.pone.0255167)
Supplement: Attachment — Submitted filename: response_to_reviewers.pdf [file pone.0255167.s002.pdf]

Reviewer #1:

This is a well written manuscript about a tool that will be a valuable addition to the scientific community. However, the choice of the single domain protein to identify the protein protein interactions is not a great one. There are other well established PPI proteins that can be used to emphasize the tool's efficiency. This will make the reader clear as to the tool's motive and its relative performance with other existing tools for PPI.

Thank you for the very kind review. We did add a case study for an additional (more "conventional") PPI complex. This was also in part per the request by Reviewer #2. Specifically, we focus on the correlation of the network differential analysis (NDA) of the efficiency centrality and some experimental data from mutation experiments that try to identify amino acids that are key in holding/establishing the stability of the protein complex. Figure 5 was added in this context as well. The added part is also highlighted in the manuscript.

Reviewer #2:

Thank you for the kind review. Please find our answers to your remarks below.

Remarks:
1. please, check intall instruction in distributive of plug-in: several files, that is necessary to download from https://www.lfd.uci.edu/~gohlke/pythonlibs/ are absent for Windows. I could not install plug-in in Windows system, Linux – OK.

Thank you for the remark. Yes, the specific version names do probably change with time, so it was not wise to refer to particular version(s). We now say more generally, to use the find function on the page to look for libraries by name prefix. We added another method worth trying if the pre-compiled libraries don't work. In general, the objective is to get PyMOL running on your computer.

2. I did not like organisation of manuscript. Many figures that are necessary for understanding the text are located in Supported material. This material are additional material, and the manuscript must be clear without them.

Well, the SI file can contain also some longer discussion of results. Some figures there have longer captions and are "two column" wide figures – to be more detailed (larger). If you specify which figure should, in your opinion, be in the main text, we can add it, but adding more than one or max two figures is perhaps not really feasible. Editors may not like too long papers in some cases :) Also, we added Figure 5 to comply with your remark #4.

3. to illustrate the work of plug-in "de novo synthesized TIM barrel protein [84]" was used. What PDB code of it?

Thank you – it is the PDB ID: 5BVL. We have added it to the text. (it can be seen in the directory name and the README file in the examples, but yes, it is much better if it is in the manuscript text, as it is now)

4. analyzing this protein, several amino acid residues have been identified as important residues for protein-protein interaction. Are there any experimental data that support this conclusions?

For this particular system there is no experimental data (that we would be aware of). Nonetheless, most of the methods implemented in the pyProGA code were already published and tested. Hence the goal of this paper is not to re-asses/re-evaluate all the methods, but to provide a user interface to them. The already public methods are FMO (with binding energy separation), the 3D SPIE – which is a visualization aid, and the SVD analysis of the PPI interface. The new technique is the NDA (network differential analysis). We do compare the results of NDA to the predictions of the FMO binding analysis and the SVD analysis to the

point where we find that they yield comparable outcomes. Hence we consider NDA to be validated against the other methods.

Nevertheless, we do acknowledge that presenting a case where the predictions of our numeric analysis could be referenced against experimental data may help the article to be more attractive for potential applicators of these methods. Hence we decided to add a short section before the Conclusions (incl. Figure 5) doing this. The added part is also highlighted in the manuscript. Please see also the response to Reviewer #1.

Minor remarks:

1. please clarify caption for fig. 3

Thank you. We have now clarified/extended the caption.

2. page 5: check link to Figure.

Assuming the link at pp. 5 on line 188 – this reference links to the caption of Figure 1 on pp. 3 (after line 101). (At least on our computers.) There is no figure shown, but this is complicit with the journals' requirement not to place the figures into the manuscript.

3. Add link to program where prmtop file can be obtained.

The prmtop file is part of the distribution on the GitLab download page https://gitlab.com/Vlado_S/pyproga in > pyProGA_disto_1.0 > examples > toy_examples.zip > subsystem > PIE-PRN-FF

The toy examples has all kinds of input file formats (.pdb, various FMO .inp/.out and .prmtop etc.). These are meant to be small, so users can orientate themselves in them and actually inspect them visually (for larger molecules the files become too big for users to realistically make sense of the data). These examples are meant to teach what is needed to build each type of PRN models, not to be physically/biologically important themselves.
